# The Comparison of Two Challenging Low Dose APIs in a Continuous Direct Compression Process

**DOI:** 10.3390/pharmaceutics12030279

**Published:** 2020-03-20

**Authors:** Tuomas Ervasti, Hannes Niinikoski, Eero Mäki-Lohiluoma, Heidi Leppinen, Jarkko Ketolainen, Ossi Korhonen, Satu Lakio

**Affiliations:** 1 School of Pharmacy, Faculty of Health Sciences, University of Eastern Finland, 70210 Kuopio, Finland; hannes.niinikoski@gmail.com (H.N.); jarkko.ketolainen@uef.fi (J.K.); ossi.korhonen@uef.fi (O.K.); 2 Orion Pharma Oyj, 02200 Espoo, Finland; Eero.Maki-Lohiluoma@orion.fi (E.M.-L.); heidi.leppinen@orionpharma.com (H.L.); satu.lakio@nanoform.com (S.L.)

**Keywords:** Continuous direct compression, low-dose API, tablet, material properties

## Abstract

Segregation is a common problem in batch-based direct compression (BDC) processes, especially with low-dose tablet products, as is the preparation of a homogenous mixture. The scope of the current work was to explore if a continuous direct compression (CDC) process could serve as a solution for these challenges. Furthermore, the principle of a platform formulation was demonstrated for low dose tablets. The combination of filler excipients and the API in the formulation used was suitable for direct compression, but also prone to induce segregation in BDC process. The CDC process was found to be very promising; it was shown that tablets with the desired quality parameters could be manufactured successfully with both of the APIs studied. Powder analysis indicated that the APIs display some fundamental differences in their physical properties, which was also reflected in powder mixture properties and, hence, eventually in processing. However, process parameters, especially mixer impeller speed, were not found to have any significant influence on end product quality. The study suggests that a CDC process can be a viable solution to resolve the challenges described. Moreover, manufacturing by using a universal platform formulation seems to be a feasible way for producing low-dose tablets.

## 1. Introduction

Especially in low-dose solid dosage form manufacturing, it is essential to ensure a homogenous distribution of active pharmaceutical ingredient (API) in order to guarantee adequate end product quality. The difficulty of spreading a small amount of API evenly into a large mass of excipients is a commonly recognized issue; a decrease in the amount of API usually increases the variability of blend potency [1,2]. Adequate mixing is obviously the first critical step, but not a guarantee for a good blend quality, since the handling of the mass after the mixing process might cause segregation, i.e., de-mixing of materials [3,4,5,6,7,8,9]. The most critical reasons for segregation are differences in the physical properties of particles, such as size, density and morphology [8,10,11,12,13,14,15,16]. When manufacturing tablets in a batch-wise manner, transporting powder mixture in containers or loading the mixture into the tablet press from a container represent very favorable circumstances for segregation. Because segregation cannot usually be detected until there is uniformity of content testing, this may lead to the rejection of the whole batch as a consequence of this undesirable phenomenon.

For about one decade, continuous manufacturing (CM) has been a hot topic in the field of pharmaceutical manufacturing. Pharma companies have invested not only in research lines but also in manufacturing lines, and as a result, at beginning of 2020 there were several FDA- and EMA-approved products on the market which were being manufactured by CM. However, the number of products is only a tiny fraction of the total amount of marketed oral solid dosage products. One reason why the pharma industry is still so strongly based on batch manufacturing might be that the best strategies to apply CM processes in pharmaceutical manufacturing are still rather unclear, i.e., for what types of products does it offer the greatest advantages? The challenges in manufacturing of low-dose products with batch processes might be one example where CM processing could be beneficial; the amount of material being processed in CM line can be kept very low, and there is no need to transfer large powder blends vertically when the powder could be in free fall (for example during transfer from a container into the tablet press), both decreasing the risk of segregation during processing [5,17,18]. Pneumatic transports, that also include free falls, are needed in certain cases in CM processing, but here also the tendency for de-mixing could be avoided by transporting only small quantities of blend within one cycle. In a direct compression process, there is, however, no need for any conveyors.

Continuous mixing has been shown to be capable to produce more homogenous mixtures as compared to batch mixing [8,19,20,21,22], thus, a continuous direct compression (CDC) set-up, where the amount of material that is being processed at a certain timepoint could be minimized by small mass hold-ups, is a promising method for low-dose manufacturing. Recently, encouraging results were obtained by Karttunen et al. (2019) when they compared a CDC process to traditional batch manufacturing [23]. The possibility of equipping the manufacturing line with suitable process analytical technology (PAT) tools, enabling the monitoring of API concentration during processing (and capability to reveal if problems are about to occur), makes it even more attractive [24].

One interesting possibility inherent in CM is the ability to develop a universal excipient formulation platform to be used for several different low-dose products with similar release mechanisms. As the API amount is low, the influence of API properties on the process and tablet quality should not be significant, thus the basic formulation could be kept constant, while only the actual drug would be changed. Therefore, major time savings could be achieved during the process and formulation development phase. Nonetheless, even though the amount of API is low and the effect on process and end product might be only minimal, some process optimization might still be needed. The questions are, how much do process settings need to be changed, what physical properties of the API are the most significant with respect to processing, and can those be forecast?

The feeding phase of raw materials has been recognized as one of the most critical process phases in several studies [25,26,27,28,29,30,31,32], especially with low-dose feeding, but also mixing must be optimized for each formulation. For example, a detailed examination of a CDC process conducted by Van Snick et al. (2017) showed the importance of process optimization for low-dose drug-products [33]. Moreover, the formulation must be developed so that it will work well in a CDC process. The good flow properties of raw materials provide easier processing, but on the other hand, they are typically prone to segregation [8,13]. A good formulation for a CDC process should have sufficiently good flow properties, but it should also be resistant against segregation.

Suitable excipients for direct compression are widely available providing a good basis for formulation development. APIs are commonly more problematic: due to demands on solubility, the particle size of the API typically has to be small and quite often micronized or even nanonized powders are used [34]. In addition, the morphology of API particles can be irregular and impossible to modify. These factors obviously worsen the flow properties of the powder, making the powders challenging to handle in continuous processes. Especially with respect to low-dose products, the API’s properties might cause serious limitations in the development of continuously manufactured tablet products. On the other hand, small particles are typically adhesive, thus the homogeneity of the mixture can be enhanced by exploiting the adherence of API particles onto the surfaces of larger particles (ordered mixture) [35]. For example, He et al. (2013) have demonstrated that an interaction between an API and excipient particles may decrease the risk of segregation due to differences in particle size [36]. Mao et al. (2013) have also reported the benefits of an ordered mixture in low-dose tablet manufacturing [37]. One challenge here is that the energy during mixing must be high enough to allow possible agglomerates of API to break down spreading particles evenly onto the mixture.

If one wishes to find a balance between sufficient flow properties for API and excipients and blend homogeneity, it is important to appreciate how different parameters affect the processability. Consequently, studies have been published describing the effect of raw material properties on mixing behavior [19,20,21,33,38,39,40]; furthermore, the interaction between material properties and process parameters with different types of continuous manufacturing set-ups has been discussed [22,24,29,33,40,41,42,43,44]. However, as far as we are aware, the number of studies where both a constant excipient formulation and excipient/API relation have been examined in a CDC process is very limited. Thus, in this study, a simple formulation was generated with two excipients with good flow properties, to see if they would have favorable characteristics to permit continuous processes. Two different APIs with slightly different physical properties and challenging flow properties were selected to estimate their effect on the process. The intention was to enable the prediction of processing behavior based on a priori measured material properties and to evaluate how well these can be balanced by different process settings (mixer impeller speed and/or total feed rate). A low dose formulation was chosen not only to demonstrate the universal low-dose formulation platform but also to highlight the possibility of adopting a direct compression process to simplify the manufacturing set-up, which is also one important goal when a process is being developed in a real life scenario. Flowability, primary particle size, possible agglomerate formation, the tendency to form an ordered mixture and the effect of continuous feeding/mixing on material properties were topics of interest.

## 2. Materials and Methods

### 2.1. Materials

Two different model APIs were intended to be formulated using the same tablet platform. The APIs used were paracetamol (Anqiu Lu’an pharmaceutical Co., Ltd., Anqiu, Shandong Providence, China) and spironolactone (Zhejiang Shenzhou Pharmaceutical Co., Ltd., Xianju, Zhejiang, China). Both APIs were chosen due to their challenging physical properties and they were recognized to be poorly flowing and, furthermore, spironolactone was known to form agglomerates. The API concentration was constant 3% (*w/w*) in all of the experiments. Microcrystalline cellulose (MCC 14% (*w/w*), Avicel PH-101 NF, FMC International Health and Nutrition, Wallingstown, Little Island, Cork, Ireland) and lactose monohydrate (LMH 82% (*w/w*), Tablettose 80, Meggle Pharma, Wasserburg, Germany) were used as fillers in the formulation and magnesium stearate (MgSt 1% (*w/w*), Ph. Eur. grade) as a lubricant.

### 2.2. Methods

#### 2.2.1. Continuous Manufacturing Line

The continuous manufacturing line was constructed of four twin screw loss-in-weight feeders (three K-ML-D5-KT20 and one K-CL-SFS-MT12, Coperion K-Tron, Niederlenz, Switzerland), a continuous mixer (Modulomix, Hosokawa Micron, Doetinchem, The Netherlands) and a rotary tablet press (PTK-PR1000, PTK CO, Ltd., Incheon, Korea). The equipment configuration was a top-down set-up, where the feeders and the mixer were located on a table over the tablet press. Thus, transporting of the powder mixture was based on gravitation so that the use of conveyors could be avoided, minimizing unknown variables and the risk of segregation. A sketch about the CDC set-up used in the study is included in the supplementary material (Supplementary Figure S1).

The larger feeders (K-ML-D5-KT20) were used for feeding of APIs, LMH and MCC while MgSt was fed with the microfeeder (K-CL-SFS-MT12). API, LMH and MCC were fed into the first inlet, and MgSt to the second inlet of the continuous mixer. The structure of the mixer has been described by our group in a previous article [29].

The feeders and the mixer were adjusted and controlled by an in-house built LabView-based software, and data from the feeders were acquired for later analysis.

#### 2.2.2. Design of Experiments

A full factorial Design of Experiments (DoE) was created for systematical evaluation of the influence of process parameters on end-product quality (Table 1). The DoE was constructed of a total of three factors/variables: total feed rate and mixer impeller speed were continuous variables with three levels, and API was a categorical variable.

Settings for each feeder were determined to meet the total feed rate (12, 20, 28 kg/h) defined in the DoE to achieve the defined ratio of API and excipients. The impeller speeds of the continuous mixer in test runs were 300 rpm (Froude number 6.4), 700 rpm (Fr 35), 1100 rpm (Fr 87). The calculation of Froude number has been described by the authors in a previous article [29].

#### 2.2.3. Feeding and Mixing

During all of the test runs, the formulation was kept constant (LMH 82%; MCC 14%; API 3%; MgSt 1%). Between the test runs, the feed rate for each feeder was adjusted so that the proportion of each material was fixed, together delivering a total mass feed rate according to Table 1. In addition, the mixer impeller speed was changed according to DoE.

Data provided by the feeders were exploited for calculating the estimated API concentration (*EC*_API_) with Equation (1):

(1) $$EC_{API}\left(t\right)=\frac{FR_{API}\;\left(t\right)}{FR_{API}\left(t\right)+FR_{LMH}\left(t\right)+FR_{MCC}\left(t\right)+FR_{\begin{array}{c} MgSt \end{array}}\left(t\right)}$$

where FR describes the total feed rate of material x at time point *t*. The *EC_API_* was compared to label claim (LC) (3% *w/w*) to estimate how accurately the desired API level was maintained during feeding.

#### 2.2.4. Tableting and Sampling

In tableting, the die filling depth was adjusted based on pre-test runs (executed with the middle point settings: 20 kg/h feed rate, 700 rpm mixer speed), to obtain the goal of a 400 mg tablet weight for both excipient–API combinations separately. Compactability curves were also plotted for both API formulations, and tablet thickness (corresponding to compaction pressure) was adjusted to obtain tablets with a breaking force around 100 N. During the actual test runs with the same API (N1–N11, N12–N22), the tablet press settings were kept constant; when the API changed, both fill depth and main compression were adjusted to the desired values.

The turret speed of the rotary tablet press was set to meet the total feed rate: the initial values for runs with 12, 20 and 28 kg/h total feed rates were 31, 52 and 73 rpm, respectively, with 16 stations. The powder level in the hopper of the tablet press was monitored visually during the runs and minor adjustment of turret speed was done manually when necessary. Force feeder speed was set to be 1.5 times the turret speed. However, in the runs with the highest turret speed, the upper limit for the force feeder speed was reached and the maximum speed of 100 rpm was used.

All powder blend samples were collected at the end of the process from the force feeder of the tablet press. Tens of tablet samples were collected according to a pre-specified sampling plan at different timepoints. The tablets for analysis were selected randomly from the collected samples.

#### 2.2.5. Powder Properties

##### Particle Size Distribution

Particle Size Distribution (PSD) of APIs were measured to detect possible changes in particle size due to feeding process. The APIs were measured without any processing and after feeding through a continuous feeder. Measurements were conducted by using a laser diffractometer (Mastersizer 2000, Malvern Instruments Ltd., Worcestershire, UK) equipped with a dry dispersion unit (Scirocco 2000, Malvern Instruments Ltd, Worcestershire, UK). API samples were measured using disperser air pressures of 0.1 and 2.0 bar to detect possible agglomerates with the lower pressure and primary particles with the higher pressure. The measurement cycle of each sample contained six replicates.

##### Powder Rheology

An FT4 Powder Rheometer (Freeman Technology, Tewkesbury, UK) was used to measure the parameters describing the flow properties of APIs and powder blends. The measured parameters were specific energy (SE) and permeability (pressure drop over the powder bed). SE is the energy required to establish a particular flow pattern in a conditioned, precise volume of powder [45]. The energies measured are mostly dependent on the inter-particulate forces and are less influenced by other factors, such as compressibility. Permeability is a measurement of how easily a material can transmit air through its bulk. A constant air flow was 5 mm/s in this study due to the appearance of dusting when using 10 or 7 mm/s through the powder bed whilst being consolidated at increasing normal stresses. Generally, a higher normal stress results in a higher pressure drop as the permeability is reduced due to particles becoming more closely packed [45]. All tests were carried out using 48 mm accessories in a 50 mm bore, borosilicate glass vessel. Pre-conditioning was done with a rotating blade in order to have a standardized initial packing condition for all samples. Measurements were done in duplicate.

##### Scanning Electron Microscopy

SEM images of the powder samples were used to obtain a deeper insight into the effect of mixing on API’s properties, and to observe the presence of agglomerates. In the imaging, a field emission scanning electron microscope (FESEM) (Carl Zeiss Sigma HD VP, Carl Zeiss, Oberkochen, Germany) was used. Before SEM photography, the samples were sputter-coated with gold using an automatic coater (Agar Auto Sputter Coater B7341, Agar Scientific Limiter, Stansted, England). In addition to SEM images, an Energy Dispersive Spectrometer (EDS), (Thermo Noran NS7 double-EDS 60 mm2, Thermo Fisher, Waltham, US) detector was utilized to determine if the API was coating the excipient particles.

#### 2.2.6. Measurement of Tablet Properties

##### Tablet Dimensions and Tensile Strengths

The dimensions of the tablets were measured using a micrometer (Digitrix, Fowler & NSK Co., Tokyo, Japan). The breaking force of the tablets was measured using a universal tester (CT-5 Tester, Engineering Systems, Nottingham, United Kingdom). The dimensions and breaking forces were used to evaluate the tensile strength, *σ*_0_ (Equation (2)) [46]:

(2) $$\sigma_{0}=\frac{2P}{\pi Dt}$$

where *P* describes applied load, *D* tablet diameter and *t* tablet thickness.

##### Tablet Friability

A friability test was carried out for every run using the Ph. Eur. tablet friability apparatus (Erweka TA3, Erweka Apparatebau GmbH, Heusenstamm, Germany). In the test, a sample of ten tablets was weighed and rotated 100 times in the apparatus. After rotating, the tablet sample was weighed again to evaluate the loss of mass.

##### Content Uniformity

A sample of six tablets from every run was taken and each tablet was weighed and dissolved in 50 mL of solvent in a volumetric flask. The solvent used was USP phosphate buffer (pH 5.8) for paracetamol tablets and 50% (*V/V*) ethanol in water for spironolactone tablets. After the tablets had dissolved, the solution was left to stand for one hour to allow the insoluble excipients to sediment. A one-milliliter sample was taken from the clear fraction of the solution and transferred to a 20 mL flask, which was filled to the mark with the same solvent to make a 1:20 dilution of the sample. The API concentration from the diluted sample was measured in a spectrophotometer (UV-1800, Shimadzu Suzhou Instruments Mfg. Co., Ltd., Suzhou, Jiangsu, China) using 242 and 243 nm wavelengths for spironolactone and paracetamol, respectively.

##### Disintegration Time

The disintegration time test was carried out for six tablets from each run. The apparatus used was USP and Ph. Eur. type disintegration apparatus (DT3, Sotax, Basel, Switzerland).

##### Dissolution

The USP dissolution test for three tablets from each run was carried out using a paddle apparatus (AT6, Sotax, Basel, Switzerland). The methods used were accordance with those described in the monographs for spironolactone and paracetamol tablets. In the spironolactone test, the medium was 0.1 N hydrochloric acid with 1% sodium dodecyl sulphate, volume 1000 mL, temperature 37 °C, paddle speed 75 rpm and testing time 30 min. In the paracetamol test, the medium was USP phosphate buffer pH 5.8, volume 900 mL, temperature 37 °C, paddle speed 50 rpm and testing time 60 min. Dissolution tests were completed at sampling time points of 4 and 16 min for paracetamol and 5 and 23 min for spironolactone. A 5 mL sample was taken from the dissolution chamber at 0.5, 2, 5, 12, 23, 39 and 60 min from the start for spironolactone tablets and at 0.5, 2, 4, 8, 16 and 30 min from the start for the paracetamol tablets and with the sample volume replaced with 5 mL of dissolution media. The samples were filtered through a 0.45 µm syringe filter and the API concentration was measured in the spectrophotometer described earlier. The timepoints when samples were taken were different for spironolactone and paracetamol for practical reasons.

The dissolution endpoint concentration was also compared to the API% measured from the tablets that were collected at the timepoint T20 (i.e., 20 min after starting the tablet press).

#### 2.2.7. Data Analysis

SAS software (SAS Institute Inc., Cary, NC) was used to construct the DoE. MODDE (v. 12, Umetrics Sartorius-Stedim Biotech, Malmö, Sweden) was used for the evaluation of data. All models were fitted with multiple linear regression (MLR). The parameters used to test the model performance were R^2^ (correlation coefficient) and *Q*^2^ (test set validation coefficient).

## 3. Results

### 3.1. API Properties

#### 3.1.1. SEM

**Figure 1:**
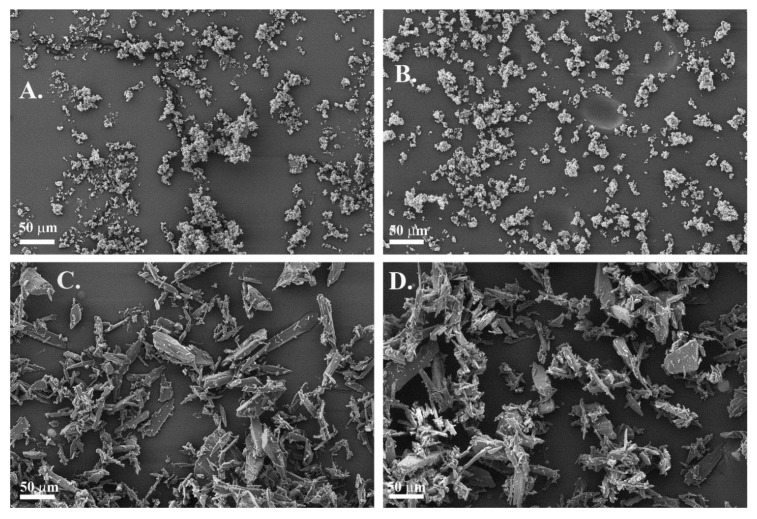
SEM images of spironolactone (**A**,**B**) and paracetamol (**C**,**D**). Unprocessed raw materials in figures (**A**,**C**) and materials fed through the feeder in figures (**B**,**D**), magnification 500×.

According to the SEM images (Figure 1A,B), the morphology of spironolactone particles revealed that they were spherical, small particles that were agglomerated together. Primary particles had more or less a constant particle size. The SEM images indicate that the feeding reduced the amount of large agglomerates in the spironolactone powder.

The morphology of paracetamol particles was typical for paracetamol: irregular, needle/plate-like, sharp particles that formed some aggregates (Figure 1C,D). According to SEM images, the paracetamol particles and their size did not change during feeding. Thus, based on the SEM images, spironolactone and paracetamol differed from each other according to their agglomeration tendencies.

#### 3.1.2. Particle Size Distribution

**Figure 2:**
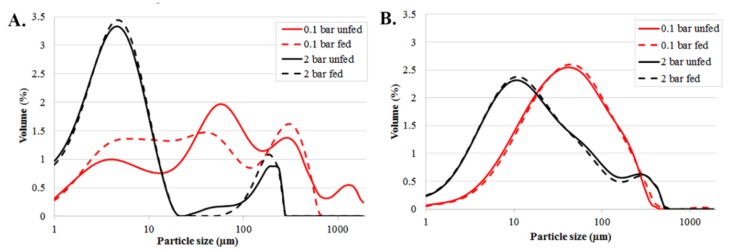
Particle size distribution of (**A**) spironolactone and (**B**) paracetamol.

Figure 2 illustrates the particle size distributions of initial raw material and material that has been fed through the feeder for both APIs.

As shown in Figure 2A, increasing the dispersing pressure affected the particle size distribution of spironolactone by increasing the amount of the smaller fraction (black vs. red lines), which could be an indication that some primary particles had slightly adhered together, i.e., agglomerated, forming secondary particles. A high pressure disintegrated the agglomerates, which could be noted as a rise in the large peak between one and 10 µm while the peaks above 50 µm had decreased. With paracetamol, a similar type of peak shifting could be noted in Figure 2B, but the difference was not as apparent as with spironolactone. This indicated that there was a clear difference between these two APIs with respect to agglomeration, supporting findings from the SEM images.

The importance of proper settings on the measurements is also highlighted by these results. There was more variation in the measurements performed using dispersing air pressure at 0.1 bar for unfed spironolactone, some variation when using 0.1 bar for fed spironolactone and very little variation when using 2.0 bar and unfed material and finally practically no variation when using 2.0 bar and fed material (data not shown). This also indicated that agglomerates were dispersed to primary particles when using a higher air pressure during the measurement.

When using the 0.1 bar of dispersing pressure, the effect of feeding on particle size could also be evaluated (Figure 2A red dash line vs. red solid line). An interesting difference in the PSD curves between the unfed and fed spironolactone lies between 40 and 190 µm. The largest peak at about 60 µm in the curve of unfed spironolactone decreases, clearly making the curve flatter, but also values at below 60 µm, i.e., the fraction below 60 µm, was higher. The peak at 350 µm increased slightly, and the peak at 1000 µm disappeared completely. Thus, feeding affected the PSD of spironolactone: the typical particle size of agglomerates was above 60 µm (peaks at 60, 350 and 1000 µm), and feeding seemed to disperse the agglomerates as the particle size decreased.

As already mentioned above, with the formation of the paracetamol agglomerate was not as apparent as with spironolactone based on PSD measurements, as the pressure change did not change the result as extensively (Figure 2B red lines vs. black lines). In addition, when the PSD of unfed paracetamol was compared to material that had been fed through the feeder (Figure 2B (dash lines vs. solid lines), no significant difference could be detected with either dispersing pressures. However, measurements where the lower pressure was used, resulted in higher particle sizes, indicating that either some agglomerates could be present that are broken down when using a higher air pressure, i.e., the primary particle disintegrated when using the higher air pressure or that particles aligned differently when the air pressure was different. When examining the SEM images, it can be seen that the primary particle size of paracetamol was unchanged, and no clear evidence of agglomeration was noted. Thus, it seemed that slightly needle-shaped particles might have aligned differently in the air flow when using different dispersion pressures. There was very little variation in all of the paracetamol PSD results (results not shown).

#### 3.1.3. Powder Rheology Measurements of APIs

There were clear differences between APIs on specific energy (SE) and permeability (Table 2). SE describes powder flow in an unconfined space. Thus, a high SE value can be an indication of interlocking and/or friction, i.e., cohesion, between particles. Spironolactone seemed to have poorer flow in unconfined space than paracetamol, most probably due to cohesion. Permeability describes a powder’s resistance to air flow. The low pressure drop refers to high permeability (air escapes easily) and can be relevant, for example during compression in tableting as air needs to be able to leave the powder bed. Permeability can also be an indicator of cohesion. Spironolactone had a significantly higher pressure drop, indicative of higher cohesion, as compared to paracetamol. In conclusion, spironolactone seemed to have poorer flowability due to cohesion compared to paracetamol according to FT4 measurements. It is important to note that, due to the low API content in the formulation, the flow properties of the powder blends are much more relevant in determining the end quality of the tablets, as long as the API can be fed steadily from the feeder, and these results are presented in the next chapter.

### 3.2. Powder Blends

#### 3.2.1. Powder Rheology Measurements of Powder Blends

The batches (N4, N5, N7, N10, N16, N18, N20, N21) for specific energy (SE) and permeability measurements were selected so that extremes were chosen in relation to high and low shear in the mixer (Table 1). The repeatability of the measurements (*n* = 2) within each run was good (results not shown), and therefore no more replicate measurements were performed. The results are shown in Table 2.

**Figure 3:**
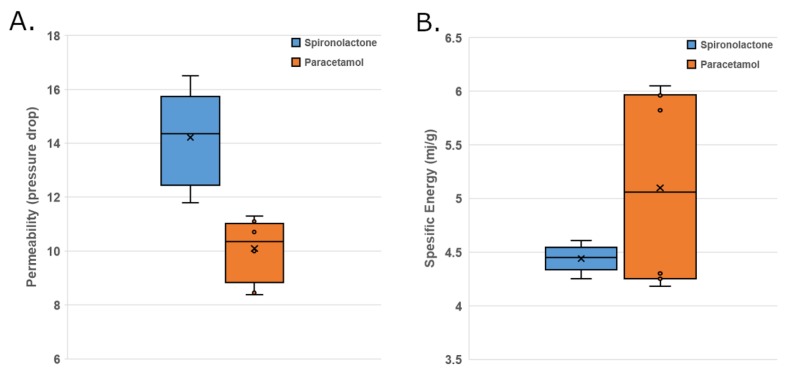
Flowability of powder blends. (**A**) permeability and (**B**) Specific energy.

According to the MLR model (*R*^2^ = 0.57, *Q*^2^ = 0.24), when the feed rate decreased SE increased with both API blends. Even though the MLR model was not statistically significant (most likely due to the extensive variation in paracetamol blends), it can be used to evaluate differences between blends. Most of the blends that had paracetamol in the formulation had higher SE values than spironolactone blends. That could be considered somewhat surprising, since pure paracetamol had a lower SE than spironolactone. However, the bulk powder blend properties can be very different as compared to pure API properties, especially when the drug load is low. It was also notable that the blends containing paracetamol had significantly higher variation with each other when compared to spironolactone blends (Figure 3). The extensive variation could indicate a higher risk for segregation or problems in gravitational flow, for instance, in die filling, if a force feeder is not used.

**Figure 4:**
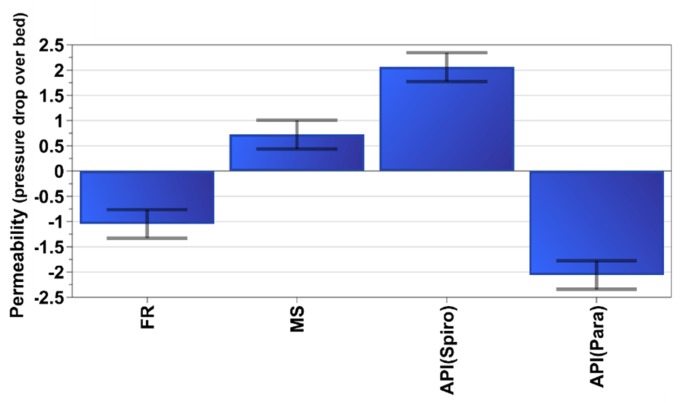
Coefficient plot for permeability. FR = Feed rate, MS = Mixer impeller speed.

According to the MLR model (*R*^2^ = 0.97, *Q*^2^ = 0.94, confidence level 95%), the most significant differences between spironolactone and paracetamol blends were evident in permeability (pressure drop). The samples containing paracetamol had lower permeability values (Figure 4) which was also consistent with the pure API permeability measurements (Table 2). In addition, a low feed rate decreased permeability, whereas a high mixer impeller speed increased it. In both of these scenarios (low feed rate or high mixer impeller speed) there was less powder in the mixer at the time (mass hold-ups will be discussed later). When there was less powder in the mixer, the powder was more fluidized, and the particles made less contact with each other. Generally, a higher normal stress resulted in a higher pressure drop, as the permeability was reduced due to particles becoming more closely packed.

When examining the permeability results in detail, a similar trend could be seen between DoE runs with both APIs: N5 and N18 (28 kg/h and 300 rpm) have the lowest permeability of their group. DoE runs N10 and N21 (28 kg/h and 1100 rpm) were next, but also with the runs N4 and N16 (12 kg/h and 300 rpm) the value was almost at the same level. N7 and N20 (12 kg/h and 1100 rpm) had the highest permeability. These results were logical, since with a high total feed rate and a low mixer impeller speed, the permeability was low, whereas with a low feed rate and a high mixer impeller speed, it was correspondingly high. One interesting finding was that with spironolactone in runs N4 (12 kg/h and 300 rpm) and N10 (28 kg/h and 1100 rpm), the pressure drop values were more or less the same as those encountered with paracetamol in runs N16 (12 kg/h and 300 rpm) and N21 (28 kg/h and 1100 rpm) respectively: this highlighted the importance of combining the fill rate of the mixer (i.e., total feed rate in this case) and mixer impeller speed in order to have a similar mass hold in the mixer.

#### 3.2.2. Homogeneity/Feeder Data

**Figure 5:**
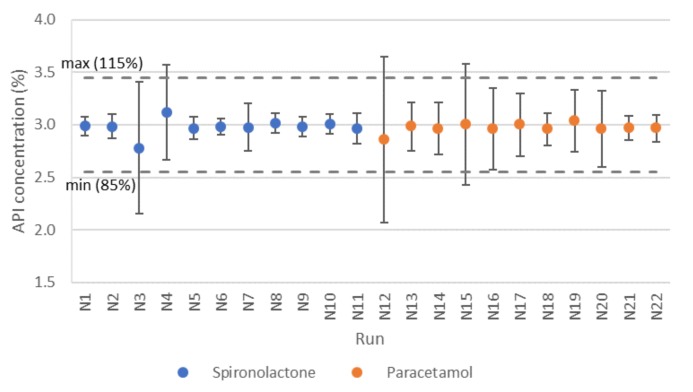
Calculated mean API concentration for each run with specification limits (85–115%).

Evaluating the uniformity of powder blends was started by comparing the data collected from the feeders during processing. The average API concentration with standard deviation (SD) were calculated over the whole run (20 min), with the results presented in Figure 5.

The average values were very good with both APIs, but the SD values of paracetamol seem to be somewhat higher. For spironolactone, runs N3 and N4 can be pointed out as having the highest variation, and run N6 can be used as a reference for a successful run with low variation (Figure 5).

More detailed research on the feeding data of run N3 revealed that the accuracy had been rather good during the run, but after 1200 s (20 min), API feeding had practically stopped (Supplementary Figure S2A). This was most likely due to the bridging of the material in the feeder (Supplementary Figure S3). The feeding data of run N4 revealed a greater variation in feeding accuracy (Supplementary Figure S2B). The total feed rate was lower in N4, and thus the feed rate of API had been lower (0.36 vs. 0.6 kg/h) which partially explained the difference. It is worth noting that even though the SD values were stated to be high for these two runs, the feeding accuracy was rather good at steady state: if the end of the N3 feeder data, and correspondingly the beginning of the N4 data, had been excluded (assuming that this material would have been discarded), then the SD values would have been notably better, probably at the same level as in run 6 (Supplementary Figure S2C), which was chosen as an example of good feeding accuracy based on SD.

In the case of paracetamol, the feeder data of run N12 exhibited a rather large variation in API feeding (Figure 5 /Supplementary Figure S2D). A closer exploration of the data revealed that, similarly as encountered with spironolactone, high SD values in the feeding accuracy of both the runs N12 and N15 could be partially explained by a rather high deviation during the start-up phase before steady-state had been reached (Supplementary Figure S2D,E), but, even after start-up, the variation in API feeding was noted to be rather high and very constant. In run N18, the variation was clearly lower but similarly constant, thus this seemed to be a common factor in all the test runs where paracetamol was used (Supplementary Figure S2F). One obvious explanation could be the impeller rotating inside the feeder that caused two similar deviations per rotation.

When comparing the feeder data of spironolactone and paracetamol runs, this kind of constant variation could not be found with spironolactone. This highlights the importance of material properties for processing linked to the particle/powder properties of these two APIs (spironolactone being more cohesive while paracetamol was more adhesive). Even though these kinds of challenges should be tackled primarily by more careful feeder selection, here the variation was still small enough that the mixer could smoothen it so that end product quality was not altered significantly (results shown later). As reported before, a mixer was capable to smoothen out the short-term fluctuations in feeding [26], depending on the residence time distribution of the specific mixer type [27,47,48,49].

#### 3.2.3. SEM Imaging and EDS Analysis of Powder Blends

SEM and EDS analysis were executed to determine if API particles were coating-excipient particles, forming an ordered mixture. However, from SEM images (Supplementary Figures S4–S6), it was very difficult to distinguish the initial API particles, and as the amount of the chemical element used as the tracker in EDS was very low in both APIs (spironolactone S, paracetamol N), and moreover as the API concentration was very low, the accuracy of these methods was not found to be good enough for reliable analysis (results not shown).

#### 3.2.4. Mass Hold

Mass hold-up of the mixer was monitored by carefully collecting all the residual material from the mixer outlet chute after shutting down the mixer. The results logically followed mixer speed, and, especially with a high mixer speed, also the total feed rate. The results are described in the supplementary material (Supplementary Figure S7).

### 3.3. Tablet Properties

#### 3.3.1. Tablet Weight and Weight Variation

The die filling depth (i.e., tablet weight) was adjusted based on pre-test runs before tableting and was kept constant in all the test runs. Thus, average tablet weights can be analyzed to describe the flow properties of powder mixture. Average tablet weights and RSDs are shown in the supplementary material (Supplementary Figure S8).

All tablets met the Ph. Eur. specification limit (±5%) with respect to tablet weight variation. The highest tablet weights for spironolactone samples were found with runs N2 (12 kg/h and 700 rpm) and N7 (12 kg/h and 1100 rpm), and the lowest with N5 (28 kg/h and 300 rpm) (Supplementary Figure S8). N7 had also the highest permeability values, while N5 had the lowest (Table 2). N4 and N10 had rather similar permeability values, but the average weight of tablets for N4 was notably higher. This was due to the different feed rate: the turret speed for N10 was higher, thus the die filling time was shorter. Because of this, only experiments that had the same process speed (i.e., total feed rate) could be compared with each other. With this approach, it seemed that there was a correlation between tablet mass and permeability, however, as the permeability values were not measured in all experiments, a comprehensive correlation comparison between tablet mass and permeability could not be done.

**Figure 6:**
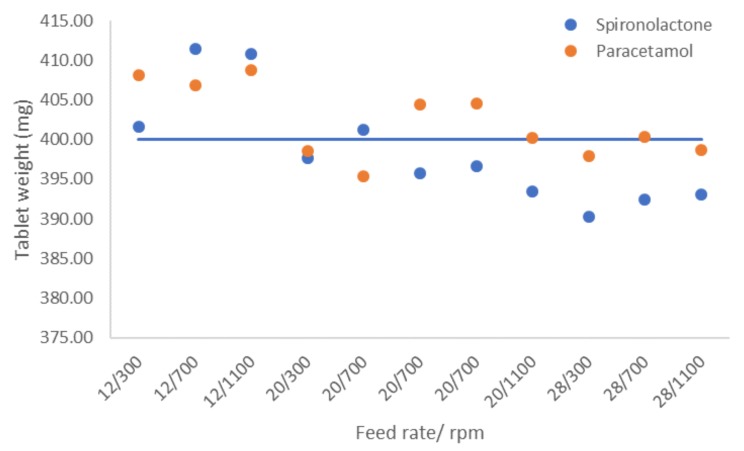
The effect of total feed rate (i.e., turret speed in tablet press) and mixer speed on tablet weight.

The effect of total feed rate (i.e., turret speed in tablet press) and mixer speed on tablet weight is presented in Figure 6. As can be seen, with a total feed rate of 12 kg/h, tablet weights are higher when compared to 20 and 28 kg/h. With spironolactone at a low feed rate (12 kg/h), the mixer speed appeared to influence the tablet weight; 300 rpm resulted in lower tablet weights than 700 and 1100 rpm (Figure 6). Interestingly, no such dependency was found in the other cases. With a total feed rate of 20 kg/h, no clear trend in the average tablet weight related to the mixer speed could be detected (Figure 6).

With a high total feed rate (28 kg/h), the average weight of paracetamol tablets did not notably change as compared to a total feed rate of 20 kg/h, which was surprising, especially as the force feeder speed did not increase linearly according to turret speed (force feeder speed was set to be 1.5 times turret speed, but, with 28 kg/h, the upper limit for the force feeder speed was reached and thus a maximum speed of 100 rpm was used instead of 109.5 rpm). With spironolactone, however, based on the data in Figure 6, it seemed that increasing total feed rate decreased the tablet weight.

Overall weight variation (within all experiments) was slightly smaller for paracetamol tablets than spironolactone tablets (Supplementary Figure S8). However, within the experiments, the variation (RSD) was ultimately very similar for both spironolactone and paracetamol tablets. Paracetamol displayed a larger variation in SE measurements that could indicate a higher risk for segregation or fluctuation in die filling, inducing the weight variation of tablets. The comparison of center point runs could support this proposal, i.e., with spironolactone, the difference between the minimum and maximum tablet weights was slightly lower (5.52 mg) as compared to paracetamol (9.12 mg). However, it seems that, with this study line set-up, which included a force feeder, we were able to avoid these issues since the weight variation in tablets was very similar for both APIs.

According to the MLR model (*R*^2^ = 0.78, *Q*^2^ = 0.65, confidence level 95%), the feed rate negatively affected tablet weight with both APIs. When looking at the model for each API separately, the effect was more pronounced with spironolactone (Supplementary Figure S9). This was a somewhat surprising result, as the API amount was so low (3%), but it still notably affected the result. However, both the SE and permeability values measured for pure APIs and mixtures support this finding, indicating poorer flow properties for the spironolactone formulation. Mixer impeller speed was not a significant factor for the tablet weights nor was there any interaction between the feed rate and mixer impeller speed.

#### 3.3.2. Tensile Strength and Friability of Tablets

The tensile strengths of spironolactone tablets were lower (average 1.5 MPa) than paracetamol tablets (average 2.2 MPa) due to spironolactone’s poorer compaction properties (results not shown). However, the friability was within the specification limit (<1%) for both APIs (spironolactone average: 0.68%, paracetamol average: 0.49%), and therefore the spironolactone tablets were sufficiently durable.

The variation in tensile strengths and friability was very small and similar for both APIs. When tensile strength values were compared as a function of total feed rate/mixer speed combination, a similar trend could be found to the tablet weight described in the previous chapter (Supplementary Figure S10).

According to the MLR model (*R*^2^ = 0.97, *Q*^2^ = 0.95, confidence level 95%) feed rate negatively affected the tensile strength of tablet with both APIs (Supplementary Figure S9). When examining the model for each of the APIs by themselves, the effect was more pronounced with spironolactone. The mixer impeller speed was not a significant factor for the tablet weights, neither was there any interaction between the feed rate and mixer impeller speed. The feed rate and mixer speed did not play any role in determining the friability of the tablets according to the MLR model, but spironolactone had a positive effect whereas, for paracetamol, it was a negative effect, i.e., spironolactone tablets had a higher tendency for friability as compared to paracetamol tablets (Supplementary Figure S9, Table S1).

#### 3.3.3. Uniformity of Content

**Figure 7:**
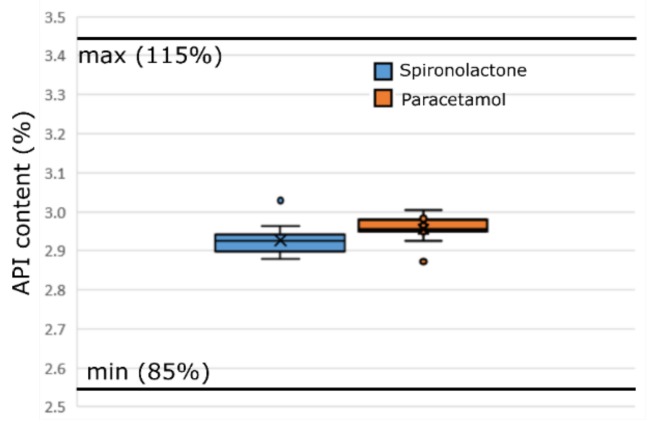
Box plot of API contents with specification limits.

All tablets met the uniformity of content specification limit (85–115%) (Figure 7 and Supplementary Table S1). Spironolactone tablets had a slightly lower content but, according to the MLR model, the difference was not significant.

The API content measured from the tablets was continuously slightly under the calculated average concentration (Supplementary Table S1). Possible reasons for this could be inaccuracies in feeding, material sticking to metal surfaces during processing and the accuracy of the analytical method.

The variation in the API concentration was slightly lower when moving from 8 to 20 min, but not remarkably. However, this could indicate variability in the API feeding, which was stabilizing towards the end of the run, and also that the conditions inside the continuous mixer were stabilizing (walls covered). As can be seen from (Supplementary Table S1), the individual values were between 2.80–3.05% which can be considered to be a very good result.

According to the MLR model, neither the feed rate nor the mixer impeller speed influenced the content. Thus, the overall homogeneity of the mass was very good with both of the APIs.

#### 3.3.4. Disintegration Time

All of the tablets met the criteria for disintegration (<15 min). The average time for spironolactone tablets to disintegrate was 39 s, and for paracetamol tablets 46 s (Supplementary Table S1). Paracetamol tablets had a larger variation, but this did not have any significance since the overall disintegration time was so short. Furthermore, according to the MLR model, there were no significant factors for disintegration.

#### 3.3.5. Dissolution

**Figure 8:**
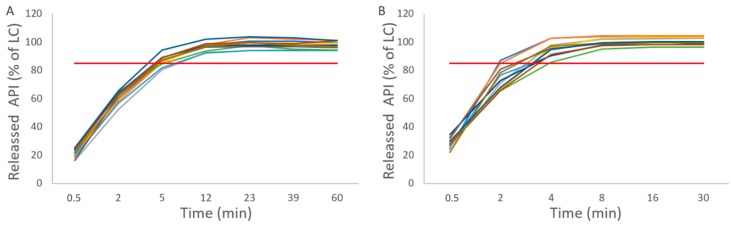
API release from tablets. (**A**) Spironolactone tablets and (**B**) Paracetamol tablets. Release for 85% limit marked with red line.

All the tablets passed the USP dissolution test. For all the tablets, dissolution was fast: over 85% of the label claim was fulfilled with paracetamol tablets at the 4 min timepoint and with spironolactone samples at less than the 12 min timepoint, respectively (Figure 8A,B). As expected, paracetamol dissolved faster due the chemical properties of the APIs rather than differences in the tablet properties. For the same reason, a shorter dissolution test with different sampling timepoints was used for paracetamol.

## 4. Discussion

One of the principal reasons for this research was to investigate the possibilities of continuous manufacturing in its simplest form, continuous direct compression, in pharmaceutical manufacturing with a challenging formulation that typically could cause problems, such as segregation, with a traditional batch manufacturing process. It was evaluated with a typical formulation prone to induce segregation in a batch direct-compression process by utilizing a formulation with a low API concentration, and excipients with good flow properties (which also are very favorable for direct compression). This demonstrates one dilemma in direct compression and continuous processing: raw materials with good flow properties must be chosen, and this usually introduces a high risk for segregation. At the same time, the API used might have very poor flow properties. In this study, by using two different types of APIs, the effect of material properties on processing could be nicely presented and at the same time, we examined the possibility of using a general formulation where only the pharmaceutical ingredient initially would be changed.

From that point of view, this study was successful and the results were very promising. The different physical properties of these APIs could be revealed by the chosen measurements, and the effect of these differences could also be illustrated for processability. With respect to the feeder data, the variation was different with both APIs, but in all cases, variations were such that the mixer could smoothen out the variations as the tablets passed the final content uniformity test. This is in good accordance with the results already presented for mixer smoothing of brief variations in feeding [29,43,44,50]. One limitation in this study was the mixer used, as it is working basically all the time in the fluidized mixing mode (i.e., a high Fr number) limiting the possibility of comprehensively demonstrating the effect of mixing parameters on end product quality. For research considerations, it would have been more interesting to use a mixer with wider possibilities for adjusting parameters (longer residence time distribution, larger hold-up), as here, no clear effect of mixer speed could be detected. However, with spironolactone, some indications about the importance of finding a balance between the total feed rate and mixer speed could be attained with low feed rates. On the other hand, the mixer used here demonstrated its ability to handle the variations seen in the feeding accuracy. As already remarked, feeding has been reported to be the most crucial part of a continuous manufacturing system; this is obviously true, but a comprehensive consideration of the whole manufacturing train, especially the feeder–mixer combination, would be a preferable approach.

Defining the optimal raw material properties in conjunction with suitable methods and then collecting those in data libraries in order to provide the critical material properties have been major topics in recent research investigating CM [51]. Here, PSD analysis, SEM imaging and rheology measurements (FT4) were used with pure APIs and on part of the powder blends. In the case of pure APIs, those were found to be very useful as they could reveal some fundamental differences in physical properties, that were also reflected in the properties of the powder mixtures (SE and permeability values), and, hence, eventually in the processing. With the methods used, indications of the effect of API physical properties and process conditions on blend properties could also be noted, but the final analysis was inadequate because samples from all the test runs could not be analyzed for practical reasons.

It was interesting to exploit the SEM imaging of powder blends to study the effect of API properties on the powder blend in more detail. Thus, SEM images and EDS analysis were used to study if API particles were coating the excipient particles (ordered mixing), which might have influenced the flow properties of the powder blends and possibly have improved the homogeneity of the blend. However, the limited accuracy of both SEM imaging and EDS analysis prevented comparison of the formation of an ordered mixture. It might be feasible to use some other techniques, such as Raman spectroscopy [52], in future studies.

In addition to the above-mentioned questions, future research should explore how this CDC system can handle longer manufacturing times. Here, the number of test runs was rather large and thus the length of each run was limited (<30 min) to control material consumption and the amount of end product/waste. However, more studies with longer running times would be needed to clarify the challenges that will arise in a real manufacturing situation, such as the filling of feeders, bridging, and the possible adherence of material onto process device surfaces resulting in random declines in high concentration clumps with high API amounts [53].

Furthermore, future work should consider a topic recently discussed in several research articles in the field of continuous tablet manufacturing, i.e., the incorporation of PAT methods into process monitoring [54,55,56], especially with low-dose formulations, which was excluded from this study. NIR measurements were executed during the test runs, but unfortunately, technical issues with the instrument forced us to leave the NIR results out of this study. However, the importance of PAT in CM is fundamental, and thus it should be investigated in future research.

## 5. Conclusions

This study investigated a CDC process with a low-dose formulation. A simple universal formulation platform principle was used, in which the portions of excipients were constant, and two different challenging APIs were individually incorporated into the blend. It was assumed that, as the API concentration was low (3%), the differences in the physical properties of APIs and their possible impact on the process/end product quality could be compensated by altering the process settings. In this study, process parameters, especially mixer impeller speed, did not have any significant influence as the process was found to be very robust also with extreme settings. However, with a different line, design process parameters could exert a more significant role.

To conclude, based on the end product analysis performed here, tablets with the desired quality parameters could be manufactured successfully for both APIs. The excipient–API combination selected for this study did have the true potential to cause segregation in a traditional batch processing but, based on these results, this could be avoided by adopting a CDC process. Thus, the change in the manufacturing method from a batch to its continuous counterpart could bring benefits when handling this type of formulation. Manufacturing using a universal platform formulation seems to be a prospective method with a low API concentration, but it is necessary to stress the importance of process optimization for each formulation. Finally, these findings reveal new interesting aspects to the pharma industry as it starts to approach continuous manufacturing.

## Figures and Tables

**Table 1:** Design of Experiments for the study.

Batch	Feed Rate (kg/h)	Mixer Speed (rpm)	API
N1	20	700	Spironolactone
N2	12	700	Spironolactone
N3	20	300	Spironolactone
N4	12	300	Spironolactone
N5	28	300	Spironolactone
N6	20	700	Spironolactone
N7	12	1100	Spironolactone
N8	28	700	Spironolactone
N9	20	1100	Spironolactone
N10	28	1100	Spironolactone
N11	20	700	Spironolactone
N12	20	700	Paracetamol
N13	20	1100	Paracetamol
N14	20	300	Paracetamol
N15	12	700	Paracetamol
N16	12	300	Paracetamol
N17	20	700	Paracetamol
N18	28	300	Paracetamol
N19	28	700	Paracetamol
N20	12	1100	Paracetamol
N21	28	1100	Paracetamol
N22	20	700	Paracetamol

**Table 2:** FT4 results for pure APIs and powder blends (*n* = 2).

Sample	SE (mJ/g)	Permeability (Pressure Drop, mBar) (5 mm/s)
Spironolactone	91.55	64.6
Paracetamol	40.10	15.9
N4	4.44	14.6
N5	4.29	11.9
N7	4.53	16.3
N10	4.50	14.2
N16	4.22	10.9
N18	5.89	8.4
N20	4.28	11.1
N21	6.01	10.0

## Supplementary Materials

The following are available online at https://www.mdpi.com/1999-4923/12/3/279/s1, Figure S1: The CDC set-up used in the experiments, Figure S2: Calculated spironolactone concentrations (A–F), Figure S3: Bridging inside the spironolactone feeder, Figure S4: SEM images of powder blend from run N5 (API = spironolactone) (A) 500× (B) 1500×, Figure S5: SEM images of powder blend from run N10 (API = spironolactone) (A) 500×, (B) 1500×, Figure S6: SEM images of powder blend from run N21 (API = paracetamol) (A) 500× (B) 1500×, Figure S7: Mass hold up, Figure S8: Average tablet weights, Figure S9: Coefficient plots for tablet weight, tensile strength and friability. Figure S10: Average tensile strengths of tablets, Table S1: Tablet properties.
