# Supplementary material for: The Comparison of Two Challenging Low Dose APIs in a Continuous Direct Compression Process
Source: Pharmaceutics. 2020 Mar 20;12(3):279. doi: 10.3390/pharmaceutics12030279 (PMC7151305; doi:10.3390/pharmaceutics12030279)
Supplement: Supplementary file 1 [file pharmaceutics-12-00279-s001.pdf]

pharmaceutics

MDPI

Supplementary materials: The Comparison of Two Challenging Low Dose APIs in a Continuous Direct Compression Process

Tuomas Ervasti Hannes Niinikoski, Eero Mäki-Lohiluoma, Heidi Leppinen, Jarkko Ketolainen, Ossi Korhonen and Satu Lakio

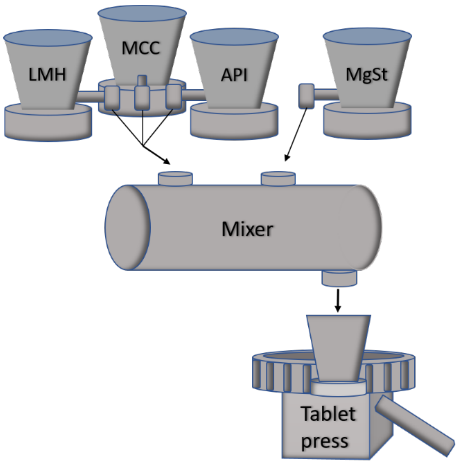

Figure S1. The CDC set-up used in the experiments.

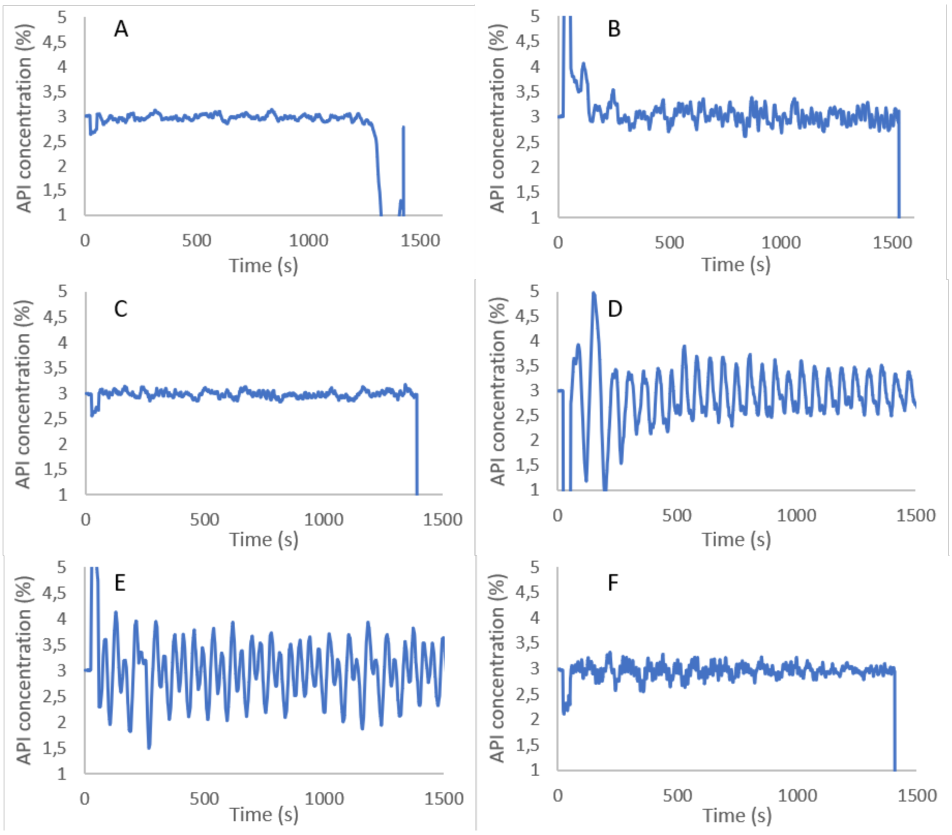

Figure S2: (**A**) Calculated spironolactone concentration (N3, 20 kg/h) (**B**) Calculated spironolactone concentration (N4, 12 kg/h) (**C**) Calculated spironolactone concentration (N6, 20 kg/h) (**D**) Calculated paracetamol concentration (N12, 20 kg/h) (**E**) Calculated paracetamol concentration (N15, 12 kg/h) (**F**) Calculated paracetamol concentration (N18, 28 kg/h).

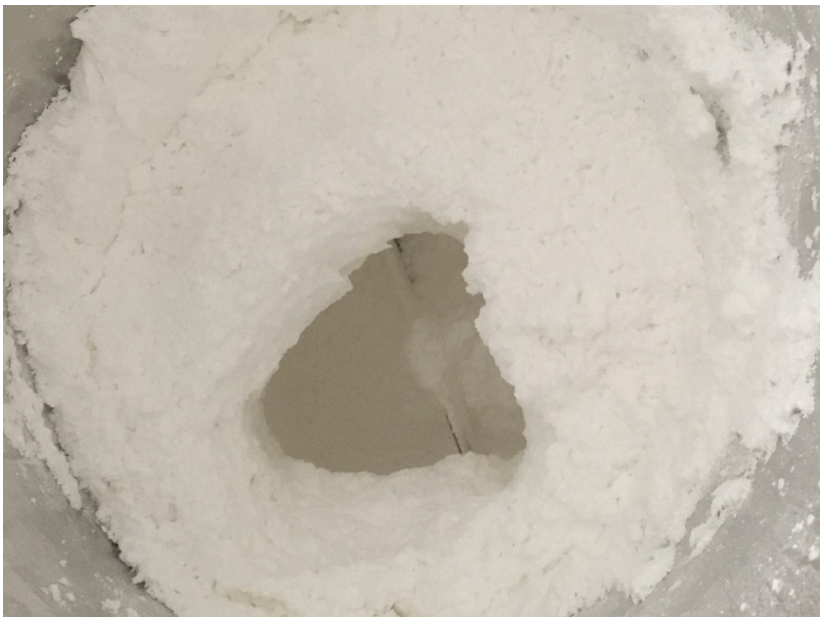

Figure S3. Bridging inside the spironolactone feeder during run N3 (20 kg/h, 300 rpm).

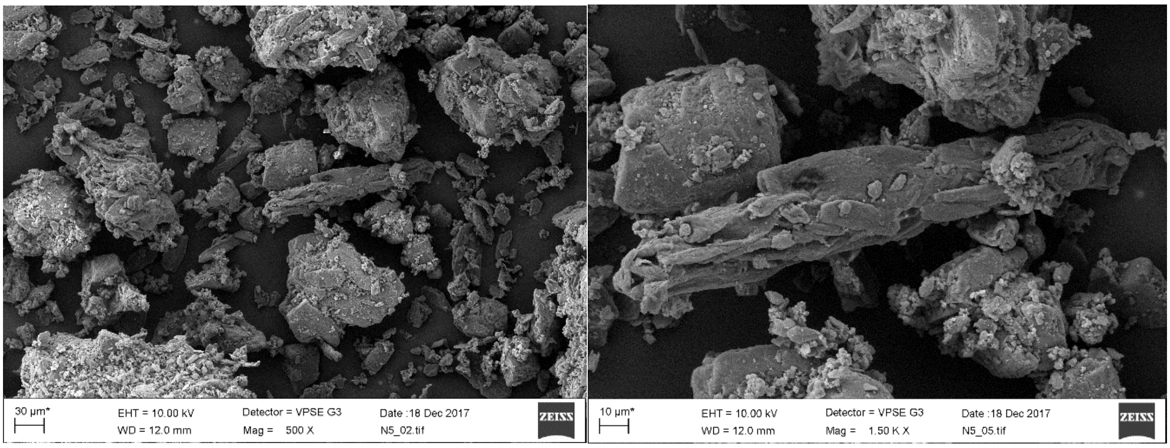

Figure S4. SEM images of powder blend from run N5 (API = spironolactone) (**A**) 500× (**B**) 1500×.

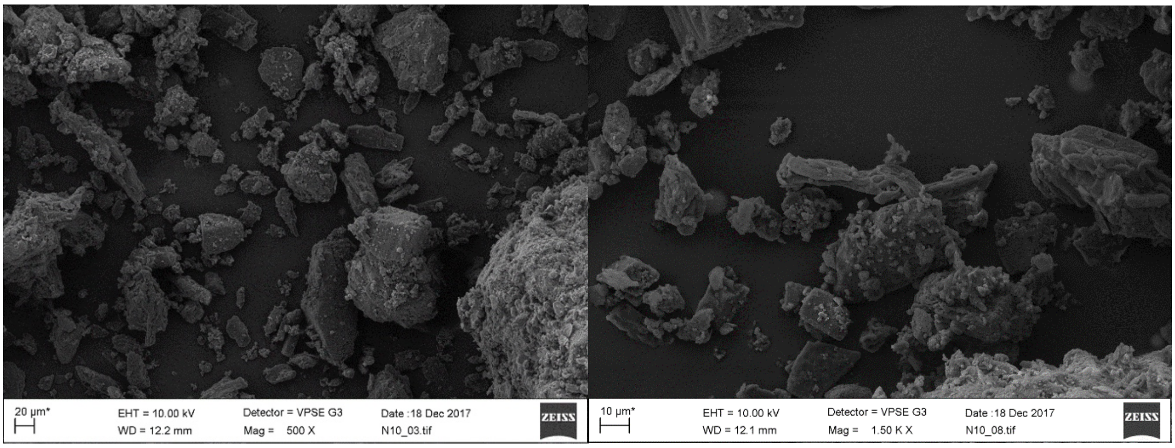

Figure S5. SEM images of powder blend from run N10 (API = spironolactone) (**A**) 500×, (**B**) 1500×.

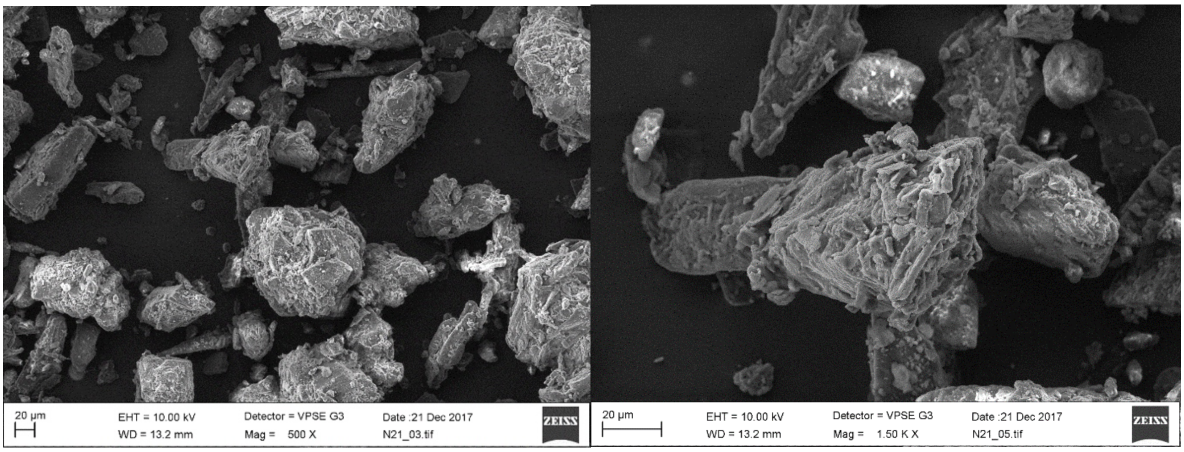

Figure S6. SEM images of powder blend from run N21 (API = paracetamol) (**A**) 500× (**B**) 1500×.

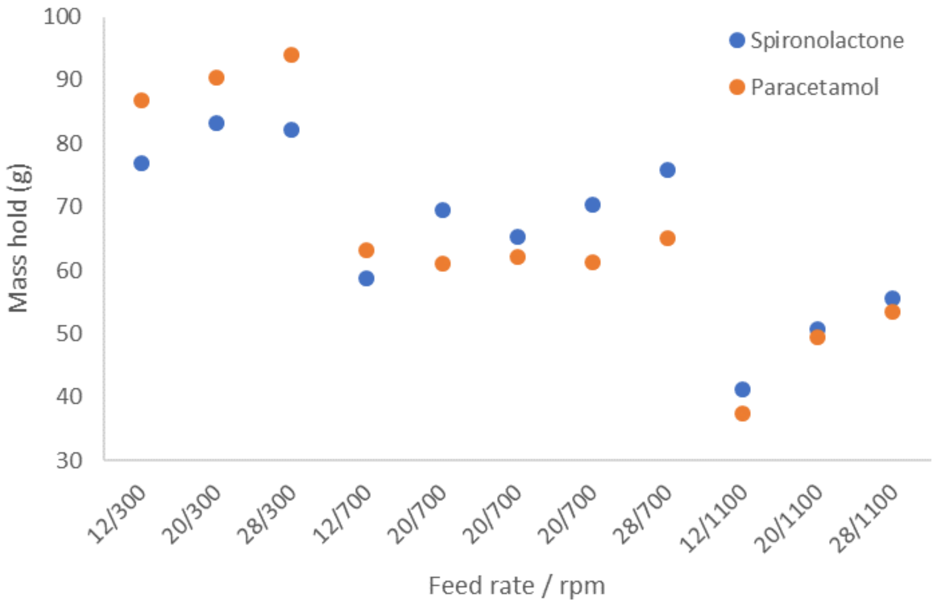

Figure S7. Mass hold up.

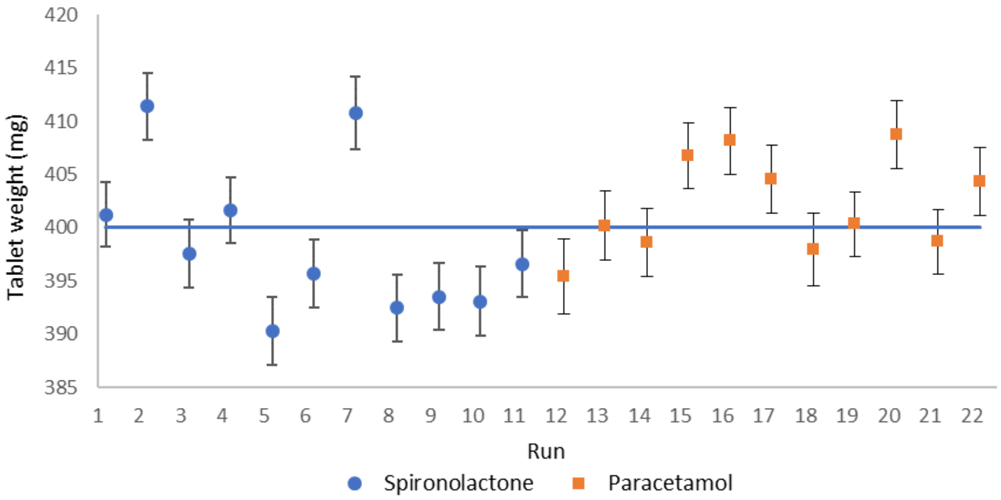

Figure S8. Average tablet weights with RSDs.

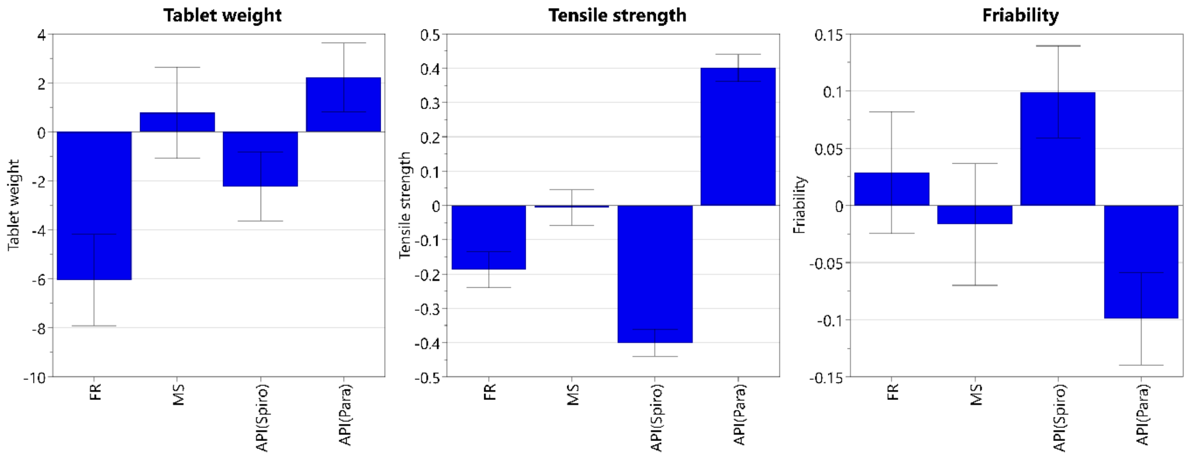

Figure S9. Coefficient plots for tablet weight, tensile strength and friability. FR = feed rate, MS = mixer impeller speed.

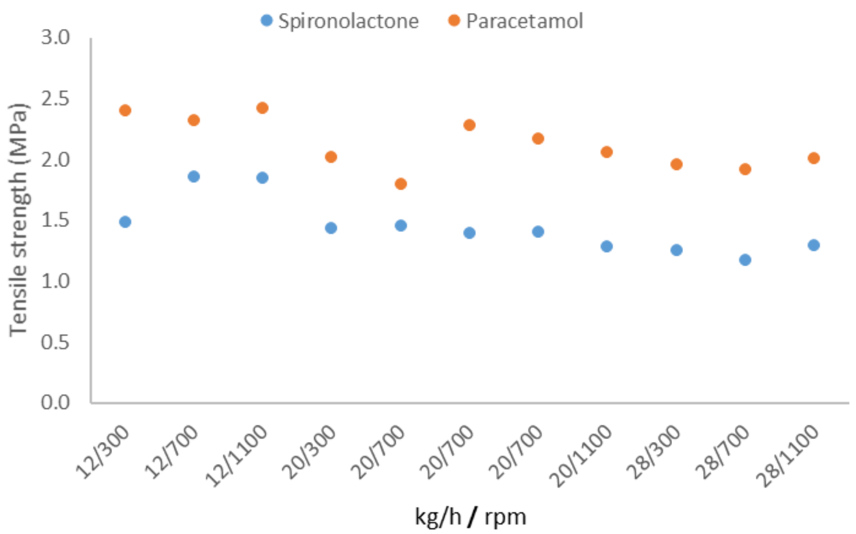

Figure S10. Average tensile strengths of tablets.

Table S1. Tablet properties.

Run	API % (@8 min)	RSD%	API% (@20 min)	RSD %	TS (@8 min)	RSD %	TS (@20 min)	RS D%	Friabil ity (%)	Dis.int. time (s)	RSD %
N1	2.92	0.45	2.90	0.60	1.41	7.01	1.51	7.57	0.71	39.3	49.9
N2	2.90	0.94	2.88	0.68	1.84	6.20	1.87	6.29	0.88	33.2	43.0
N3	2.92	1.95	2.90	1.31	1.41	9.39	1.46	7.24	0.68	44.5	75.7
N4	2.95	0.36	2.92	1.33	1.48	6.71	1.48	5.41	0.63	37.0	22.4
N5	2.94	0.67	2.90	0.63	1.23	4.78	1.28	6.33	0.71	32.2	29.8
N6	2.91	0.49	2.93	1.37	1.38	9.19	1.40	5.28	0.66	126.2	122.9
N7	2.90	0.81	2.89	0.83	1.84	6.35	1.86	8.02	0.43	38.0	8.8
N8	2.98	0.39	2.95	0.53	1.14	7.20	1.20	6.56	0.72	45.0	33.9
N9	2.96	0.33	2.96	0.40	1.19	8.26	1.37	6.38	0.73	43.2	19.6
N10	2.97	1.27	3.03	0.29	1.27	7.18	1.30	7.66	0.78	34.7	19.3
N11	3.03	0.37	2.93	0.35	1.33	8.69	1.48	12.91	0.61	43.2	31.2
N12	2.95	0.68	3.00	1.66	1.75	2.69	1.83	13.13	0.55	66.3	62.51
N13	3.01	0.57	2.98	1.00	1.95	8.55	2.16	6.07	0.47	39.0	23.56
N14	2.95	0.65	2.96	1.00	1.94	6.64	2.09	7.25	0.48	50.0	33.82
N15	2.99	0.59	2.97	0.85	2.26	4.68	2.37	5.38	0.42	46.2	32.29
N16	2.94	0.72	2.95	0.65	2.24	3.60	2.55	4.99	0.58	27.2	21.39
N17	3.00	0.57	2.87	0.68	2.31	5.28	2.23	6.46	0.44	55.8	37.22
N18	2.85	0.74	2.92	0.64	1.87	4.75	2.04	7.32	0.51	44.2	62.41
N19	2.93	0.53	2.95	0.81	1.90	6.96	1.92	7.60	0.49	42.2	32.63
N20	2.92	0.57	2.98	0.96	2.30	6.04	2.53	4.29	0.44	31.2	42.97
N21	3.05	0.37	2.97	0.68	2.02	5.13	1.98	6.25	0.52	69.7	97.53
N22	2.97	0.89	2.95	0.99	2.12	5.67	2.22	7.96	0.49	41.7	43.32

API = Active pharmaceutical ingredient, TS = Tensile strength, Dis.int.time = Disintegration time.
